# DNA Methylation in an Enhancer Region of the FADS Cluster Is Associated with FADS Activity in Human Liver

**DOI:** 10.1371/journal.pone.0097510

**Published:** 2014-05-19

**Authors:** Timothy D. Howard, Rasika A. Mathias, Michael C. Seeds, David M. Herrington, James E. Hixson, Lawrence C. Shimmin, Greg A. Hawkins, Matthew Sellers, Hannah C. Ainsworth, Susan Sergeant, Leslie R. Miller, Floyd H. Chilton

**Affiliations:** 1 Center for Genomics & Personalized Medicine Research, Center for Public Health Genomics, Wake Forest School of Medicine, Winston-Salem, North Carolina, United States of America; 2 Division of Allergy and Clinical Immunology, Department of Medicine, The Johns Hopkins University, Baltimore, Maryland, United States of America; 3 Department of Internal Medicine Section on Molecular Medicine and Section on Pulmonary, Critical Care, and Allergic and Immunologic Disease, Wake Forest School of Medicine, Winston-Salem, North Carolina, United States of America; 4 Department of Cardiology, Wake Forest School of Medicine, Winston-Salem, North Carolina, United States of America; 5 Human Genetics Center, University of Texas Health Science Center at Houston, Houston, Texas, United States of America; 6 Program in Molecular Genetics and Genomics, Wake Forest School of Medicine, Winston-Salem, North Carolina, United States of America; 7 Department of Biochemistry, Wake Forest Health Sciences, Winston-Salem, North Carolina, United States of America; 8 Department of Physiology/Pharmacology, Wake Forest Health Sciences, Winston-Salem, North Carolina, United States of America; 9 Wake Forest Center for Botanical Lipids and Inflammatory Disease Prevention, Wake Forest University Health Sciences, Winston-Salem, North Carolina, United States of America; National Cancer Institute, National Institutes of Health, United States of America

## Abstract

Levels of omega-6 (n-6) and omega-3 (n-3), long chain polyunsaturated fatty acids (LcPUFAs) such as arachidonic acid (AA; 20∶4, n-6), eicosapentaenoic acid (EPA; 20∶5, n-3) and docosahexaenoic acid (DHA; 22∶6, n-3) impact a wide range of biological activities, including immune signaling, inflammation, and brain development and function. Two desaturase steps (Δ6, encoded by *FADS2* and Δ5, encoded by *FADS1*) are rate limiting in the conversion of dietary essential 18 carbon PUFAs (18C-PUFAs) such as LA (18∶2, n-6) to AA and α-linolenic acid (ALA, 18∶3, n-3) to EPA and DHA. GWAS and candidate gene studies have consistently identified genetic variants within *FADS1* and *FADS2* as determinants of desaturase efficiencies and levels of LcPUFAs in circulating, cellular and breast milk lipids. Importantly, these same variants are documented determinants of important cardiovascular disease risk factors (total, LDL, and HDL cholesterol, triglycerides, CRP and proinflammatory eicosanoids). *FADS1* and *FADS2* lie head-to-head (5′ to 5′) in a cluster configuration on chromosome 11 (11q12.2). There is considerable linkage disequilibrium (LD) in this region, where multiple SNPs display association with LcPUFA levels. For instance, rs174537, located ∼15 kb downstream of *FADS1*, is associated with both *FADS1* desaturase activity and with circulating AA levels (p-value for AA levels = 5.95×10^−46^) in humans. To determine if DNA methylation variation impacts FADS activities, we performed genome-wide allele-specific methylation (ASM) with rs174537 in 144 human liver samples. This approach identified highly significant ASM with CpG sites between *FADS1* and *FADS2* in a putative enhancer signature region, leading to the hypothesis that the phenotypic associations of rs174537 are likely due to methylation differences. In support of this hypothesis, methylation levels of the most significant probe were strongly associated with FADS1 and, to a lesser degree, FADS2 activities.

## Introduction

Association studies have identified hundreds of relationships between single nucleotide polymorphisms (SNPs) and phenotypic traits [1]. However, only a few studies have clearly determined the causal mechanisms that underlie observed SNP-trait associations. Variants may affect gene expression in several ways, including altering the regulatory landscape (e.g., promoter or enhancer) of a gene, alternative splicing, transcript degradation, and transcription of non-coding RNA. However, associations are typically due to a genetic variant in linkage disequilibrium (LD) with the associated SNP. Alternatively, an epigenetic alteration may be responsible for the observed biological variation. Recent genome-scale mapping of DNA methylation suggests that methylation impacts gene transcription via a variety of mechanisms, depending on its location (i.e., transcriptional start sites, gene bodies, regulatory elements and repeat sequences) within the transcriptional unit [2].

**Figure 1:**
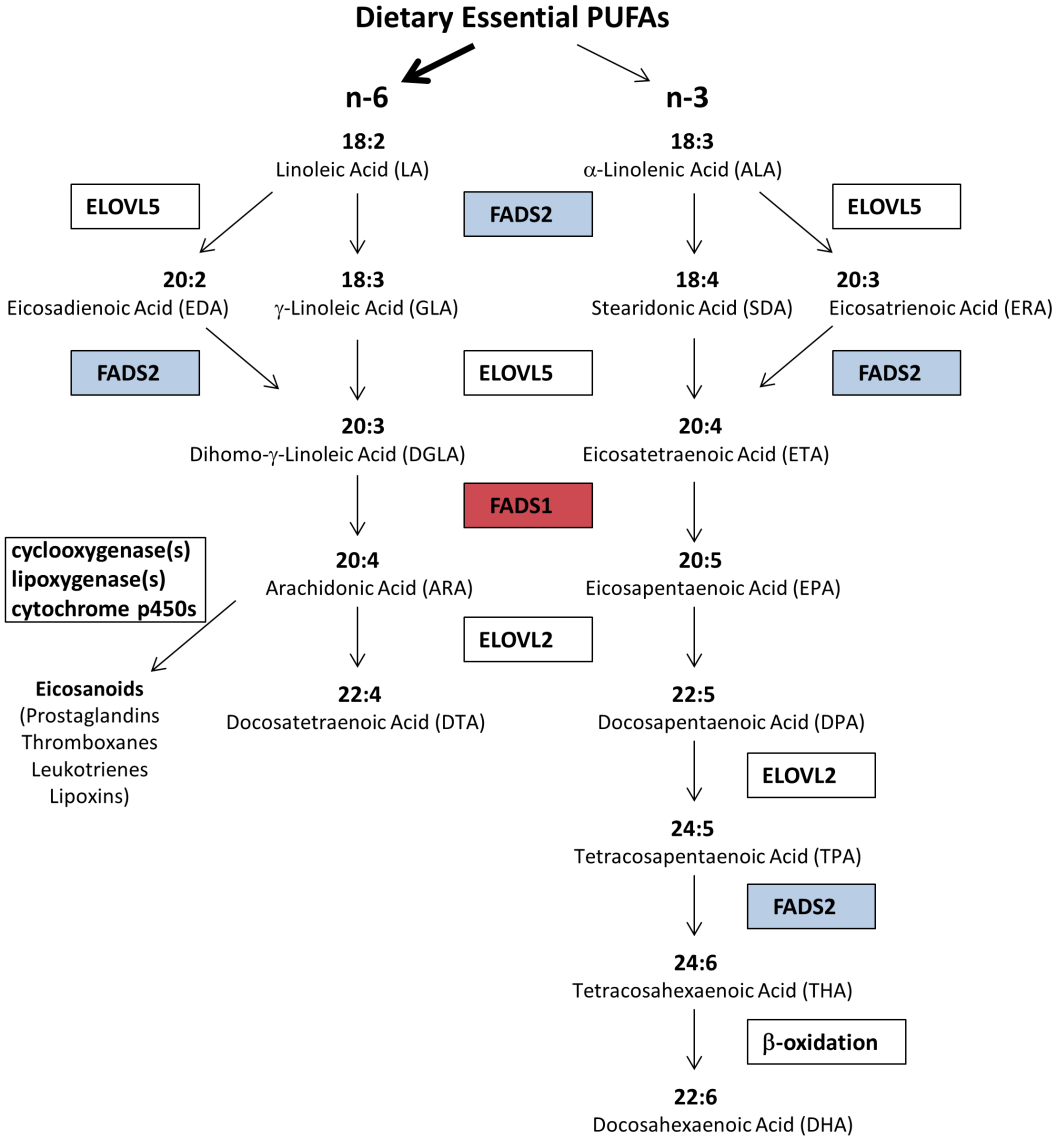
LcPUFA biosynthesis from dietary essential PUFAs Pathway. Key enzymes and metabolites are shown, and the roles of *FADS1* (red) and *FADS2* (blue) are indicated.

Long chain PUFAs (LcPUFAs) can be synthesized from essential, 18 carbon PUFAs (18C-PUFAs) found in the human diet, primarily as vegetable oil products (soybean, corn, palm, and canola oils as well as margarine and shortenings) (Figure 1). There has been a dramatic (3–4 fold) increase in the dietary levels of the omega-6 18C-PUFA, linoleic acid (to 6–8% of daily energy consumed) in the past 50 years, as well as an intense debate about the health impact of this rise [3]. Much of this discussion centers on a difference in opinion with regard to the degree that humans have the capacity to convert 18C-PUFAs to LcPUFAs. Once LcPUFAs are formed, they are then incorporated into glycerolipids found in lipoprotein particles in cells and tissues (Figure 1). Once in cellular membranes, LcPUFAs can be liberated (typically after immunologic activation) as free fatty acids by a family of phospholipases (PLA_2_), diacylglyceride, and monoacylglyceride lipases and converted to a large family of eicosanoid products (including prostaglandins, thromboxanes, leukotrienes and lipoxins) [4]. In general, these eicosanoids act like local hormones to regulate acute and chronic inflammation in numerous human diseases.

First cloned in 1999 [5], the Δ5 (*FADS1*) and Δ6 (*FADS2*) desaturases are encoded by members of a gene family cluster (*FADS1*, *FADS2* and *FADS3*) localized to a 100 kb region on chromosome 11 (11q12.2) [5], [6]. Speculated to have arisen during human evolution by gene duplication, *FADS1-3* have a high degree of sequence identity (62–70%), almost identical intron/exon organization [5] and are highly conserved between species. Numerous studies have identified associations between genetic variants in *FADS1* and *FADS2* and LcPUFA levels, as well as precursor-product desaturase activities, with little evidence for genetic loci outside of 11q12.2 (reviewed in Lattka et al., 2009 [7]). Other studies have found strong associations between variants in the FADS cluster with total, LDL, and HDL cholesterol, triglycerides, phospholipids, C-reactive protein, proinflammatory eicosanoids, and cadiovascular disease endpoints[8]–[13].

While much of the aforementioned research has been carried out in populations of European descent, recent evidence documents consistent effects in populations of African ancestry [14], [15]. The extensive LD in European Americans is considerably less in African Americans, and rs174537 is the SNP with the strongest evidence for association with AA levels [14], [16]. rs174537 is located within the 3′ end of *MYRF* (myelin regulatory factor), but is also within a haplotype block spanning ∼30 kb (based on the 1000 Genomes Project) that includes *FADS1* and coincides with the peak positive selection signal. This block overlaps completely with the peak association signal with LcPUFAs in African Americans [14], and includes rs174537 as well as three known eQTL SNPs for *FADS1* [17] (rs174547, rs174548 and rs174549). Importantly, there are dramatic differences in the frequency of the allele (G) associated with increased levels of LcPUFAs and FADS1 activity in African American populations [14], [15] studied to date, and most notably, this allele is fixed in African populations [18]. There is strong evidence across multiple sources, including the 1000 Genomes Project and the Human Genome Diversity Panel, that this fixation is likely to have occurred as a result of positive selection approximately 85,000 years ago [18].

## Results and Discussion

**Figure 2:**
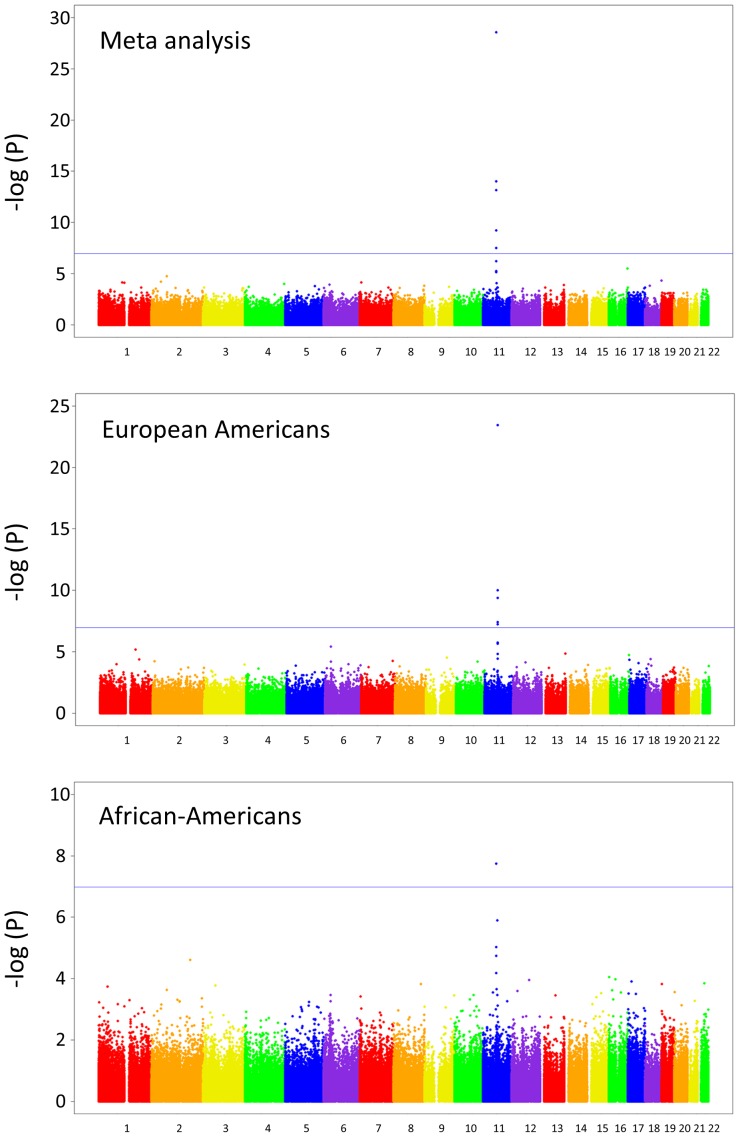
Manhattan plots for association of rs174537 with HumanMethylation450 sites. P-values are based on the genetic trend test and adjusted for age, chip, and chip position. The blue line indicates the Bonferroni-adjusted level of significance of 1.03×10^−7^ (0.05/485,000). The upper panel shows results from the meta-analysis (using European Americans and African-Americans), the middle panel shows European Americans only, and the lower panel shows African-Americans only.

Given recent evidence that variation in DNA methylation can be associated with *cis* genetic variation [19], [20], we performed a genome-wide, allele-specific methylation (ASM) analysis with rs174537. Our hypothesis was that rs174537 may serve as a genetic proxy for DNA methylation within the FADS cluster. As liver is known to be a primary organ involved in LcPUFA biosynthesis, DNA was extracted from 144 liver samples obtained from the Pathobiological Determinants of Atherosclerosis in Youth (PDAY) study [21]. Briefly, PDAY was an autopsy study designed to examine the pathogenesis of atherosclerosis in young people. A subset of the total population, selected for a separate study to examine non-HDL cholesterol, consisted of subjects with the lowest 25^th^ (controls) and highest 10^th^ (cases) percentile of non-HDL cholesterol. Samples were from 72 European American and 72 African-American males, 15 to 34 years of age, who died of violent causes within 72 hours after injury and underwent autopsy in one of the cooperating medical examiners’ laboratories. rs174537 genotypes were obtained as part of a custom genotyping panel of 77 SNPs, utilizing the Sequenom iPlex genotyping system (see Methods). To determine if ASM associations existed throughout the genome, all probes represented on the Illumina HumanMethylation450 BeadChip (485,577 CpG sites) were tested for association with rs174537. Only one region, on chromosome 11q12.2, showed a marked ASM association with rs174537 in both ethnicities; meta-analysis was performed combining both ethnicities, accounting for sample size and direction of effect (Figure 2). The most significant association was observed with the methylation probe cg27386326 (p = 2.69×10^−29^ for the meta-analysis; p = 3.66×10^−24^ in European Americans and p = 1.78×10^−08^ in African Americans), located approximately 3.5 kb from the *FADS1* transcription initiation site. As with previous association reports with rs174537 and LcPUFA levels, the effect size of rs174537 on methylation status was similar between the two groups (β = −0.19±0.01 in European Americans and β = −0.26±0.04 in African Americans). Four other sites – cg16213375 (p = 9.76×10^−15^), cg10515671 (p = 6.93×10^−14^), cg03805684 (p = 6.22×10^−10^), and cg19610905 (p = 3.09×10^−8^) – reached a Bonferroni-adjusted level of significance (i.e., 0.05/485,000 = 1.03×10^−7^; blue line in Figure 2). Validation of the original finding was performed using pyrosequencing of the cg27386326 CpG site, with the probe CG27386326_04 (Qiagen, Inc.). DNA methylation of the locus with pyrosequencing was strongly correlated (r^2^ = 0.81) with the BeadChip450 k result. To ensure that selection bias was not affecting our results in this population, we performed a simple association analysis with rs174537 and case-control status based on the non-HDL phenotype. No evidence of association was observed (p = 0.71, additive model, race-adjusted).

**Figure 3:**
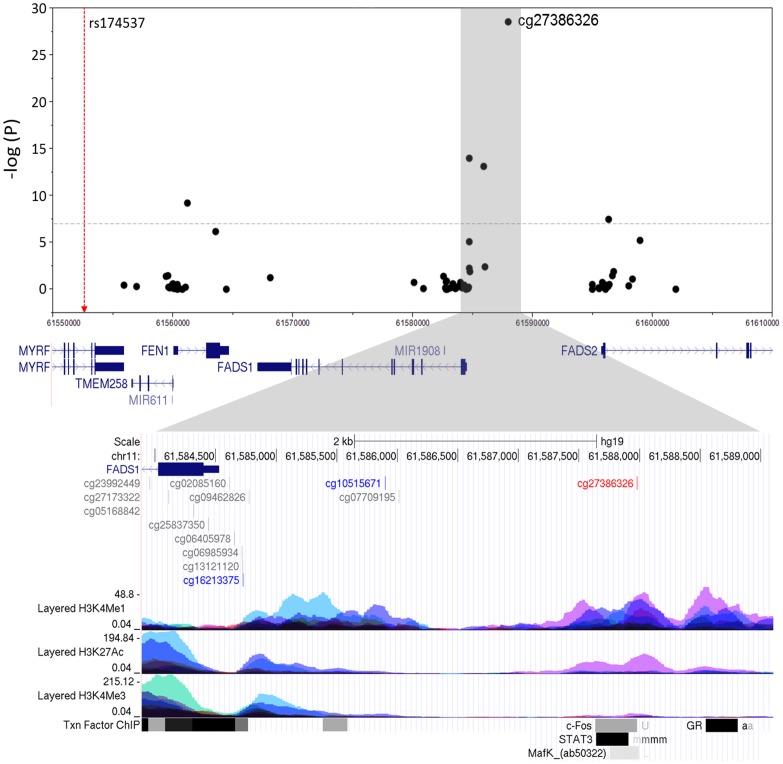
Association of *FADS* cluster CpG sites with rs174537. The lower panel shows the location of the CpG sites relative to the ENCODE data for H3K4Me1, H3K27Ac, and H3K4Me3 histone marks, respectively, which indicate promoter and/or regulatory regions in seven cell lines. The peak methylation site is shown in red, and the other two sites from the region that met Bonferroni adjustment are shown in blue.

Closer examination showed that these methylation loci are located in a likely regulatory region 5′ of both *FADS1* and *FADS2* (Figure 3). ENCODE regulation tracks revealed that the highly associated methylation region is located in a region that has a potential “enhancer signature,” namely monomethylation of histone H3 lysine4 (H3K4me1) and acetylation of H3K27 (H3K27Ac; lower zoomed portion of Figure 3). In addition, this site is in or near transcription factor binding sites for c-Fos, STAT3 and MafK.

**Figure 4:**
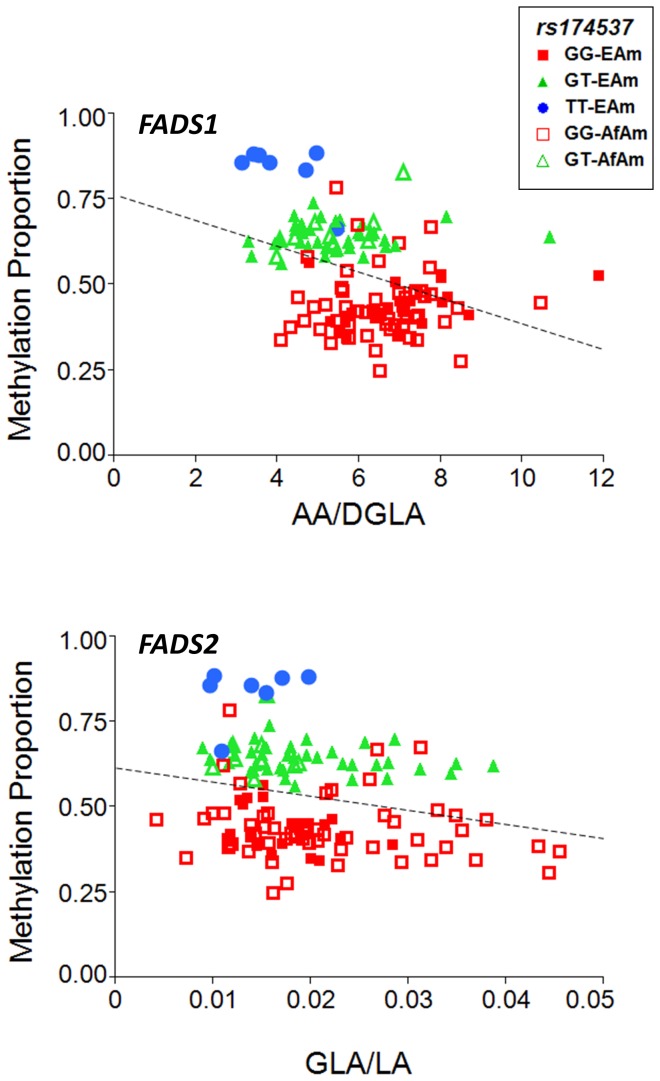
DNA methylation ratio and AA/DGLA (*FADS1* efficiency) and GLA/LA (*FADS2* efficiency) of cg27386326 stratified by rs174537 genotype. Markers for each ethnic group, by genotype, are indicated in the legend.

To better determine the putative role of cg27386326 methylation and *FADS* activity, we measured fatty acid levels within the liver samples and examined association between methylation levels and the key surrogate measures of *FADS1* and *FADS2* activities, the ratios of AA to DGLA and GLA to LA levels, respectively. There was a strong association between methylation status of cg27386326 and AA/DGLA (FADS1 activity) in liver tissues (ANOVA p = 3.99×10^−6^; Figure 4). GLA/LA (FADS2 activity) showed a more modest relationship with methylation status (p = 0.0067; Figure 4). It is worth noting that statistical differences in associations between FADS1 and FADS2 activities may center around the difficulty of approximating FADS2 activity by measuring fatty acid levels within liver tissue. Here, the newly-formed product of the FADS2 reaction, GLA, is rapidly converted to DGLA via elongation and can no longer be measured, thus underestimating its activity. In contrast, the product of the FADS1 reaction, AA, accumulates in cells and tissues such as the liver.

Figure 4 also illustrates the distribution of rs174537 genotypes among the samples, with GG, GT and TT clearly associated with different levels of methylation at cg27386326. This methylation proportion differed by 40% between homozygotes at rs174537 (44%, GG genotype; 84%, TT genotype). To our knowledge, this magnitude of allele-specific or allele-associated methylation has not been observed outside of the imprinting and X-chromosome inactivation context.

One possible explanation for the association between the genetic variant rs174537 and cg27386326 methylation level is that a SNP in LD with rs174537 exists at the targeted cg27386326 CpG site. Such a methylation-altering SNP, known as an mSNP, would lead to an apparent change in DNA methylation that was actually due to alternate genotypes at the CpG site [22]. To rule out this possibility, we sequenced the region encompassing the cg27386326 probe in the study population and found no SNP within the 50bp probe sequence or the targeted CpG site.

Both genotype and the related methylation were associated with FADS activities in the liver, but the effect of genotype nearly goes away once methylation is accounted for. Taken together, these data suggest that rs174537, and SNPs in LD with rs174537, may serve as genetic markers for the methylation status of one or more critical CpG sites in the FADS gene cluster. This possibility is made more interesting by the noteworthy location of these sites, specifically in a region between the promoters for *FADS1* and *FADS2* and within a putative enhancer histone modification signature. These data also raise the key question of whether both genes are regulated by a single mechanism, the methylation of an enhancer region between two proximal promoters.

This study has several strengths and limitations. Given the nature of the PDAY study, we have been able to examine methylation levels in a highly relevant tissue for PUFA metabolism, and correlate those levels with a SNP that has been associated in population studies. Unfortunately, gene expression levels could not be measured, due to mRNA degradation of these autopsy samples. However, a recent study analyzing eQTL in liver samples from 427 Caucasian subjects showed that rs174548, which is in strong LD with rs174537 in the CEU population (r^2^ = 0.81 in the 1000 Genomes Project) was highly associated with two probes for *FADS1* expression (p = 1.52×10^−4^ and 1.74×10^−5^) [17]. These data suggest that the impact of rs174537 on *FADS1* activity is altered via transcription, raising the important question of how methylation of this enhancer region impacts expression of genes in the *FADS* cluster. Certainly, recent studies reveal that enhancers located at variable distances from promoters can play a key role in regulating gene expression [2]. While they are typically in CpG-poor regions, whole-methylome analysis has shown them to have highly variable levels of methylation [23]. Moreover, several recent studies have linked the methylation status of an enhancer region and enhancer function, but the interaction between methylation and transcription factor binding is still poorly understood [24], [25].

**Figure 5:**
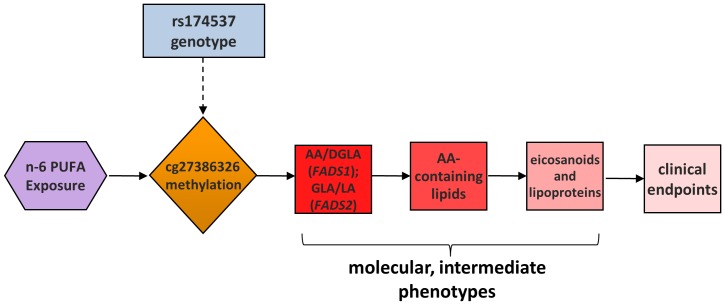
A model for a causal role of cg27386326 methylation in *FADS* activities and resulting molecular and clinical phenotypes.

We propose a pathway in which methylation of cg27386326 (and nearby loci) plays a key causal role in the formation of LcPUFA-containing lipids, cholesterol, triglyceride and eicosanoid formation, and ultimately clinical endpoints associated with human disease (Figure 5). As described above, there has been a dramatic increase in human exposure to n-6 18C-PUFA over the past 50 years. Additionally, it is clear that certain SNPs within the FADS cluster, such as rs174537, are strongly associated with the degree to which 18C-PUFAs are converted to LcPUFAs and the formation of LcPUFA-containing lipids [3], [14], [15], [18]. Perhaps it is not surprising that many of these same variants are strongly associated with biomarkers of human disease, including total cholesterol, LDL-cholesterol, HDL-cholesterol, triglycerides and eicosanoids, given that LcPUFAs play a key role in the biosynthesis of each. This study suggests that rs174537 is a strong genetic proxy for DNA methylation in a putative enhancer region of the *FADS* cluster, and that the methylation status of these loci may directly impact FADS1 and FADS2 activity.

## Materials and Methods

### Study Samples

Liver samples were obtained from the Pathobiological Determinants of Atherosclerosis in Youth (PDAY) study [21]. PDAY was an autopsy study designed to examine the pathogenesis of atherosclerosis in young people. A subset of the total population, selected for a separate study, consisted of subjects with the lowest 25^th^ (controls) and highest 10^th^ (cases) percentile of non-HDL cholesterol. Samples were from 72 European American and 72 African-American males, 15 to 34 years of age, who died of violent causes within 72 hours after injury and underwent autopsy in one of the cooperating medical examiners’ laboratories. DNA was isolated from liver samples that had been stored at −80C. Five hundred to 700 mg of thawed liver tissues were homogenized with a Dispomix Drive (Medic Tools AG, Switzerland) and genomic DNA extracted with a MagneSil Genomic, Large Volume system (Promega, USA) process that had been automated on a Freedom EVO liquid handler (Tecan, Switzerland). Extracted DNA was quantitated with PicoGreen reagent (Molecular Probes, USA) and verified as high molecular weight (>50 Kb) by agarose gel electrophoresis. This study used DNA obtained from liver samples acquired at autopsy. Since all study subjects were deceased at the time of study, use of these specimens is not considered Human Subjects research.

### SNP Genotyping

A panel of 77 SNPs for genotyping in six fatty acid candidate genes (*FADS1, FADS2, ELOVL2, ELOVL5, ACAD11 and ACOX1*) were selected by using 1) using an r^2^ threshold of 0.7 in the HapMap CEU population, and 2) additional SNPs to guarantee tagging at an r^2^ threshold of 0.7 in the HapMap YRI population, using the tagger option in Haploview [26]. rs174537 was forced to be a tagging SNP, due to its extensive associations in the literature. SNP genotyping was performed with the Sequenom iPlex genotyping system, and only rs174537 was included for analysis in this study.

### Sequencing Around the cg27386326 Probe Sequence

DNA probes on the HumanMethylation450 BeadChip consist of 50bp fragments, and the subsequent base is the assayed CpG. To exclude the possibility that the CpG site included a SNP, we sequenced the 50bp probe and immediately surrounding sequence using Sanger sequencing. A 724 bp PCR product was amplified using the following primers: 5′-ATGATGTAAGTTTGGCTACAGAGA-3′ and 5′-CAATTCAGCAAATTTATGTGGG-3′. PCR cycling conditions were 95°C, 5 min; 30 cycles of 95°C for 30 sec, 56°C for 30 sec, and 72°C for 1 min; followed by a 5 min extension at 72°C. Sequencing reactions were performed using the ABI BigDye Terminator V1.1 chemistry. Sequencing products were run on an ABI 3730×L DNA Analyzer (Applied Bioystems, Inc., Foster City, CA) and analyzed with Sequencher V4.8 (GeneCodes Corp, Ann Arbor, MI).

### DNA Methylation Analysis

Samples were evaluated using the Illumina HumanMethylation450 BeadChip, which assays 485,577 unique CpG sites. The average beta (essentially the ratio of the methylated to unmethylated signal) for each site was used to test for differences by genotype. Association analysis was performed in the European American and African-American samples separately using a generalized linear model (proc glm), as implemented in SAS (Cary, NC). Age, chip, and chip position were included in the model as covariates. Meta analysis was performed using METAL [27], weighting by sample size and accounting for direction of effect.

### Measurement of Fatty Acids in Liver Samples

Fatty acids were measured in homogenized liver samples. Tissue was homogenized in ice-cold deionized water at 100 mg/ml. Fatty acid methyl esters were prepared in triplicate homogenate samples (100 µl) following a modification of Metcalfe *et al.* [28] and analyzed by gas chromatography as previously described [29]. Fatty acids in samples were identified based on retention times of commercially available authentic fatty acid methyl esters.
